# Supplementary material for: Workshop, Assessment, and Validity Evidence for Tools Measuring Performance of Knee and Shoulder Arthrocentesis
Source: MedEdPORTAL. 2023 Apr 13;19:11309. doi: 10.15766/mep_2374-8265.11309 (PMC10101652; doi:10.15766/mep_2374-8265.11309)
Supplement: Supplementary file 1 — Shoulder Checklist and GRS.docxKnee Checklist and GRS.docxSim Case 1 - Knee.docxSim Case 2 - Shoulder.docxTraining 1 - Intro.mp4Training 2 - Knee.mp4Training 3 - Shoulder.mp4Workshop Flow.docxVisual Aid - Knee 1.pdfVisual Aid - Knee 2.pdfVisual Aid - Shoulder.pdfInjection Workflow Visual.pdfAssessor Training - Knee 1.mp4Assessor Training - Knee 2.mp4Assessor Training - Shoulder 1.mp4Assessor Training - Shoulder 2.mp4Postworkshop Survey.docx [file mep_2374-8265.11309-s001.zip › B. Knee Checklist and GRS.docx]

| **Knee Injection/Aspiration Checklist** | | | | | | |
| --- | --- | --- | --- | --- | --- | --- |
| **For Administrative Use** | **General Instructions** | | |  |  |  |
| Learner: | Circle the number of points awarded for each checklist item; some scores are all-or-nothing, and others award partial credit. Circle the number that corresponds to your global rating of the observed procedure. *Denotes major checklist items. | | | | | |
| Evaluator: |  |  |  |  |  |  |
| **Pre-Procedure** | | | | | | |
| **Checklist Item** | **Description** | | | **Score** | | |
| **(1)** Individualizes informed consent to the patient scenario* | Discusses indications, local and systemic risks, contraindications, and alternatives | | | 0 | 2 | 4 |
| **Procedure** | | | | | | |
| **Checklist Item** | **Description** | | | **Score** | | |
| **(2)** Positions the patient* | Supine with knee flexed 20° and supported or sitting with knee dangling | | | 0 | 2 | 4 |
| **(3)** Uses landmarks to mark the injection site* | -Supine medial/lateral: patellar margins. Injection site: superior third of patella  -Supine superolateral: patellar margins. Injection site: 1cm superolateral to superolateral corner of patella  -Seated: patellar margins & patellar tendon. Injection site: next to patellar tendon | | | 0 | 2 | 4 |
| **(4)** Uses sterile technique* | | | | | | |
| **(a)** Sterilizes the site | Iodine or Chlorhexadine as per manufacturer instructions | | | 0 | 1 | 2 |
| **(b)** Maintains sterility | "No Touch" technique or sterile gloves; re-sterilizes if breaks sterility | | | 0 | | 2 |
| **(5)** Performs a Time Out | Reports patient name, procedure, and site | | | 0 | 1 | 2 |
| **(6)** Anesthetizes locally | Sprays ethyl chloride or injects lidocaine along trajectory | | | 0 | | 2 |
| **(7)** Needle Driving Technique* | Aspirates while advancing needle and drives the needle towards the target based on selected approach  Supine medial/lateral: advance towards patellar midpoint  Supine superolateral: advance towards patellar midpoint  Seated: direct posterior and towards midline | | | 0 | 2 | 4 |
| **Post-Procedure** | | | | | | |
| **Checklist Item** | **Description** | | | **Score** | | |
| **(8)** Manages sharps | Does not attempt to recap needle; deploys safety cap (if present); disposes needle in a sharps receptacle | | | 0 | | 2 |
| **(9)** Cares for injection site | Applies pressure; applies a bandage | | | 0 | | 2 |
| **(10)** Individualizes post-injection anticipatory guidance to the patient scenario | Provides advice on possible systemic effects, signs and symptoms of a septic joint, signs and symptoms of a post-injection flare, icing and resting the joint | | | 0 | 1 | 2 |
| **Global Rating Score** | | | | | | |
| Unsatisfactory | | Satisfactory | Exceptional | | | |
| 1 2 3 4 5 6 7 8 9 | | | | | | |
| Learner performs unsatisfactorily, for instance lacking confidence with the procedure, demonstrating poor procedural flow, making many minor mistakes, failing to complete a major item, failing to communicate with the patient during the procedure | | Learner performs satisfactorily, for instance showing some hesitancy during the procedure, making a few minor mistakes, backtracking, communicating moderately well with the patient | Learner performs exceptionally, for instance is at ease with the procedure, demonstrating procedural flow, making no mistakes, and communicating effectively with the patient | | | |
| **Total Score** (checklist + global rating score) | | | / 39 points | | | |
