# Supplementary material for: Workshop, Assessment, and Validity Evidence for Tools Measuring Performance of Knee and Shoulder Arthrocentesis
Source: MedEdPORTAL. 2023 Apr 13;19:11309. doi: 10.15766/mep_2374-8265.11309 (PMC10101652; doi:10.15766/mep_2374-8265.11309)
Supplement: Supplementary file 1 — Shoulder Checklist and GRS.docxKnee Checklist and GRS.docxSim Case 1 - Knee.docxSim Case 2 - Shoulder.docxTraining 1 - Intro.mp4Training 2 - Knee.mp4Training 3 - Shoulder.mp4Workshop Flow.docxVisual Aid - Knee 1.pdfVisual Aid - Knee 2.pdfVisual Aid - Shoulder.pdfInjection Workflow Visual.pdfAssessor Training - Knee 1.mp4Assessor Training - Knee 2.mp4Assessor Training - Shoulder 1.mp4Assessor Training - Shoulder 2.mp4Postworkshop Survey.docx [file mep_2374-8265.11309-s001.zip › C. Sim Case 1 - Knee.docx]

**Knee Scenario**

Ms. Jones is a 67-year-old woman with a history of knee osteoarthritis who presents to clinic for a scheduled knee injection. She also has a history of well-controlled diabetes. Her last injection was 6 months ago, and it alleviated her symptoms for about 5 months. Orthopedic surgery evaluated her last year; she is a candidate for total knee replacement, but the patient prefers to delay the surgery with conservative measures as long as possible. She requests local anesthetic prior to injection.

Please complete the following:

- Consent the patient and perform a time-out
- Perform the procedure, making sure to:
  - Verbally position your assessor for the procedure
  - Verbalize your chosen approach and explain why
  - Use sterile technique
- Provide post-procedural care and instructions

This is a hybridized simulation assessment. Your assessor will serve as the standardized patient for consent, positioning, and post-procedural care. Demonstrate your sterilization, injection, and post-procedural care techniques on the mannequin.
