# Supplementary material for: Workshop, Assessment, and Validity Evidence for Tools Measuring Performance of Knee and Shoulder Arthrocentesis
Source: MedEdPORTAL. 2023 Apr 13;19:11309. doi: 10.15766/mep_2374-8265.11309 (PMC10101652; doi:10.15766/mep_2374-8265.11309)
Supplement: Supplementary file 1 — Shoulder Checklist and GRS.docxKnee Checklist and GRS.docxSim Case 1 - Knee.docxSim Case 2 - Shoulder.docxTraining 1 - Intro.mp4Training 2 - Knee.mp4Training 3 - Shoulder.mp4Workshop Flow.docxVisual Aid - Knee 1.pdfVisual Aid - Knee 2.pdfVisual Aid - Shoulder.pdfInjection Workflow Visual.pdfAssessor Training - Knee 1.mp4Assessor Training - Knee 2.mp4Assessor Training - Shoulder 1.mp4Assessor Training - Shoulder 2.mp4Postworkshop Survey.docx [file mep_2374-8265.11309-s001.zip › D. Sim Case 2 - Shoulder.docx]

**Shoulder Scenario**

Mr. Smith is a 63-year-old man who presents with shoulder pain. He has worked as a drywaller since he was 18 years old. About 5 years ago, he started noticing pain in his shoulder when working above his head. The pain improved with rest. On exam, Hawkin’s and Neer’s tests are positive. He agrees to try a steroid injection.

Please complete the following:

- Consent the patient and perform a time-out
- Perform the procedure, making sure to:
  - Verbally position your assessor for the procedure
  - Verbalize your chosen approach and explain why
  - Use sterile technique
- Provide post-procedural care and instructions

This is a hybridized simulation assessment. Your assessor will serve as the standardized patient for consent, positioning, and post-procedural instructions. Demonstrate your sterilization, injection, and post-procedural care techniques on the mannequin.
