# Supplementary material for: Workshop, Assessment, and Validity Evidence for Tools Measuring Performance of Knee and Shoulder Arthrocentesis
Source: MedEdPORTAL. 2023 Apr 13;19:11309. doi: 10.15766/mep_2374-8265.11309 (PMC10101652; doi:10.15766/mep_2374-8265.11309)
Supplement: Supplementary file 1 — Shoulder Checklist and GRS.docxKnee Checklist and GRS.docxSim Case 1 - Knee.docxSim Case 2 - Shoulder.docxTraining 1 - Intro.mp4Training 2 - Knee.mp4Training 3 - Shoulder.mp4Workshop Flow.docxVisual Aid - Knee 1.pdfVisual Aid - Knee 2.pdfVisual Aid - Shoulder.pdfInjection Workflow Visual.pdfAssessor Training - Knee 1.mp4Assessor Training - Knee 2.mp4Assessor Training - Shoulder 1.mp4Assessor Training - Shoulder 2.mp4Postworkshop Survey.docx [file mep_2374-8265.11309-s001.zip › Educational Summary Report_11309.pdf]

MedEdPORTAL® The AAMC Journal of Teaching and Learning Resources

Original Publication

Open Access

Workshop, Assessment, and Validity Evidence for Tools Measuring Performance of Knee and Shoulder Arthrocentesis

Jacob Murray, DO*, Andrea Ramirez-Gomez, MD, Mike Cahill, PhD, Amber Deptola, MD, Colin Diffie, MD, Peter McDonnell, MD, John P. Metzler, MD, Nathan P. Olafsen, MD, Lisa Zickuhr, MD, MHPE

*Corresponding author: jacob.murraydo@gmail.com

Abstract

Introduction: Musculoskeletal concerns are common, yet residents at our institution lacked arthrocentesis training. We created a workshop to teach residents knee and shoulder arthrocentesis, developed simulated assessment scenarios (SASs) with tools to measure procedural proficiency, and collected validity evidence. **Methods:** A multidisciplinary group conducted a modified Delphi to define content for the workshop, SASs, and assessment tools. We defined minimum thresholds for competence in knee and shoulder arthrocentesis using the modified borderline-group method. We implemented the workshop and SASs in 2020 and 2021 and analyzed assessment tool scoring for statistical reliability and validity. Our program evaluation included SAS performance, participants' survey responses, and change in the number of arthrocenteses performed in the internal medicine (IM) resident primary care clinic. **Results:** Sixty-one residents (53 IM, eight physical medicine and rehabilitation [PM&R]) participated. Fifty-two (85%; 46 IM, six PM&R) completed the evaluation survey. We procured data from 48 knee and 65 shoulder SASs for validity evidence. All arthrocentesis SAS performances met the proficiency standard except one resident's shoulder SAS. Validity evidence revealed strong interrater reliability ($\alpha = .82$ and .77 for knee and shoulder, respectively) and strong relational validity ($p < .001$ for both procedures). All participants rated workshop quality and usefulness as good or very good. The number of arthrocenteses performed at our institution's primary care clinic increased. **Discussion:** We created a workshop to teach residents arthrocentesis and assessment tools with strong validity and reliability evidence. The workshop was well regarded by residents, who applied their arthrocentesis skills during patient care.

Keywords
Arthrocentesis, Assessment, Clinical/Procedural Skills Training, Flipped Classroom, Internal Medicine, Primary Care, Quantitative Research

Educational Objectives

By the end of this session, learners will be able to:

1. List the indications, contraindications, risks, benefits, and alternatives for knee and shoulder arthrocentesis.
2. Demonstrate no-touch technique for knee and shoulder arthrocentesis.
3. Provide postprocedural instructions following knee and shoulder arthrocentesis.

Citation:
Murray J, Ramirez-Gomez A, Cahill M, et al. Workshop, assessment, and validity evidence for tools measuring performance of knee and shoulder arthrocentesis. *MedEdPORTAL*. 2023;19:11309.
https://doi.org/10.15766/mep_2374-8265.11309

Introduction

Musculoskeletal complaints commonly motivate patients to seek medical care.[1] Arthrocentesis is a cornerstone of evaluating and treating these problems, with the knee and shoulder being the most injected joints.[2] As such, the American College of Physicians (ACP) lists arthrocentesis as an important skill for internists to master.[3] While the clinical objectives of arthrocentesis and intra-articular injections differ, the techniques overlap. In this publication, the term *arthrocentesis* represents both procedures in accordance with ACP nomenclature.

Familiarity with knee and shoulder arthrocentesis is declining,[4] and national claims data suggest that primary care providers are performing fewer arthrocenteses.[5] Locally, internal medicine (IM) residents at Washington University in St. Louis School of Medicine (WUSM) identified gaps in arthrocentesis training during interviews at the end of the 2018-2019 academic year, and only 10 (7%) IM residents performed as few as 15 arthrocenteses

Copyright © 2023 Murray et al. This is an open-access publication distributed under the terms of the Creative Commons Attribution license.

during the 2019-2020 academic year. Reduced physician training, comfort, and performance of these procedures may delay patients' time to diagnosis and treatment.

Studies demonstrate that using models to teach arthrocentesis leads to better skill development than lecture or patient encounters alone.[5] The lack of well-developed measures for procedural competence has hindered arthrocentesis training initiatives. Most arthrocentesis skills assessments have relied on trainees' self-reported confidence levels without data demonstrating that they improve procedure quality.[6-8] The few studies that do focus on assessing procedural competence are unpublished, fail to gather validity evidence, or have produced poor-to-moderate reliability measures.[5,9-13]

Behavioral learning theory states that skills are learned through practice and feedback.[14] Following this theory, we created an educational initiative for residents to learn the skills of knee and shoulder arthrocentesis through repetition and expert guidance, as well as assessment tools with strong validity evidence to measure procedural proficiency in knee and shoulder arthrocentesis.

Methods

Content Identification

We assembled two teams of internists, physiatrists, and rheumatologists skilled in knee (Amber Deptola, Colin Diffie, Peter McDonnell, John P. Metzler, Nathan P. Olafsen, and Lisa Zickuhr) and shoulder (Dominique Cosco, Colin Diffie, Maria Gonzalez-Mayda, John P. Metzler, Nathan P. Olafsen, and Lisa Zickuhr) arthrocentesis. Each team member consulted the literature and their individual expertise to generate a list of items essential for teaching and assessing knee and shoulder arthrocentesis.[2] We gathered items and reached a consensus on which to include in educational materials through a modified Delphi method.[15] We set our consensus level as an agreement among four of six survey participants (67%). The modified Delphi results (Tables 1-3) served as a content guide for curricular and assessment materials.

Assessment Development

We used the results of the modified Delphi surveys to create assessment tools for knee and shoulder arthrocentesis comprising checklist items and a global rating score ranging from 1 to 9 (Appendices A and B). The checklist outlined critical steps in preprocedural, procedural, and postprocedural arthrocentesis care. Members of our multidisciplinary team (Amber Deptola, Colin Diffie, Peter McDonnell, John P. Metzler, Nathan P. Olafsen, Andrea Ramirez-Gomez, and Lisa Zickuhr) assigned points to checklist items through deductive conversation, drawing upon collective experience in knee and shoulder arthrocentesis. We classified each checklist item as major or minor, where major items were considered essential to the performance of knee and shoulder arthrocentesis. Minor items were thought to be important steps that might not affect the outcome of the procedure if performed incorrectly. Items related to individualizing informed consent, positioning the patient, using anatomic landmarks to identify the injection site, using sterile technique, and needle driving technique were classified as major. They were weighted twice as heavily as the minor items. This strategy assigned a cumulative point value of major items greater than the total of minor items, quantifying the essential nature of the major items within total checklist scores. We then selected items for which trainees could earn partial credit, deciding that dichotomous tasks such as maintaining sterility, managing sharps, and caring for the injection site did not warrant partial credit because learners would either complete or miss them.

The global rating score assessed the overall performance of the knee or shoulder arthrocentesis, incorporating items not referenced on the checklist. We wrote behavioral anchors to describe some components influencing global performance, such as communication skills, procedural flow, and knowledge of the procedure, providing a general depiction of unsatisfactory, satisfactory, and exceptional performances. Final assessment scores represented the sum of checklist items and the global rating score.

We wrote two simulation assessment scenarios (SASs) for knee and shoulder arthrocentesis that reflected scenes commonly encountered during patient care (Appendices C and D). The SASs prompted learners to perform skills identified in our modified Delphi. They emphasized different aspects of arthrocentesis (e.g., the effects of comorbid conditions such as diabetes mellitus on arthrocentesis informed consent) to assess transferability to residents' knowledge and skills.

Members of our multidisciplinary team (Colin Diffie, Jacob Murray, Nathan P. Olafsen, and Lisa Zickuhr) described expectations for competence in knee and shoulder arthrocentesis. We used the modified borderline-group method to define proficiency as cutoff scores on our assessment tools.[16] Lisa Zickuhr wrote hypothetical SAS performance scenarios that the team reviewed in conjunction with SAS performance data collected during the workshops. Hypothetical SAS scores ensured the representation of a variety of score distributions in the analysis. The median score for the borderline groups determined the cutoff score for the knee and shoulder arthrocentesis assessment tools.

Copyright © 2023 Murray et al. This is an open-access publication distributed under the terms of the Creative Commons Attribution license.

Table 1. Modified Delphi Results for Knee and Shoulder Arthrocentesis Pre- and Postprocedural Checklist Items

Item	Round 1		Round 2		Included or Excluded
	Total Votes	No. of Votes to Include Item	Total Votes	No. of Votes to Include Item	
Trainee can review indications pertinent to the patient, including:					
Diagnostic	8	8			Included
Therapeutic, generally	8	6			Included
Therapeutic, with specific indications (e.g., osteoarthritis, inflammatory arthritis)	8	2			Excluded
Trainee can review contraindications pertinent to the patient, including:					
Overlying cellulitis	8	6			Included
Poorly controlled diabetes or hypertension	8	2			Excluded
Uncontrolled coagulopathy	8	5	6	6	Included
Acute fracture	8	2			Excluded
Concern for septic joint	8	6			Included
Allergy to lidocaine	8	6			Included
Drug interactions with lidocaine (e.g., ritonavir)	8	2			Excluded
Prosthetic joint	8	6			Included
Anticoagulation use	8	3	6	0	Excluded
Trainee obtains informed consent, reviewing risk/benefits and providing:					
An overview of procedure	8	8			Included
An option of declining	8	8			Included
Benefit—diagnostic information	8	7			Included
Benefit—relief of symptoms	8	8			Included
Risk—infection	8	8			Included
Risk—postinjection flare	8	7			Included
Risk—fat pad atrophy	8	4	6	2	Excluded
Risk—trauma to surrounding structures	8	7			Included
Risk—bruising or bleeding	8	8			Included
Risk—skin depigmentation	8	2			Excluded
Risk—hyperglycemia	8	6			Included
Risk—headache	8	1			Excluded
Risk—pain	8	6			Included
Risk—facial flushing	8	3			Excluded
Risk—cartilage thinning	8	5	6	5	Included
Trainee gathers all necessary materials, including:[a]					
Syringes	8	7			Included
Local lidocaine	8	8			Included
Iodine or chlorhexidine	8	8			Included
Nonsterile gloves	8	7			Included
Gauze/bandage	8	8			Included
Hemostat for needle exchange	8	3			Excluded
Needles	8	8			Included
Ethyl chloride spray	8	6			Included
Trainee provides postprocedural care, including:					
Applying pressure	8	8			Included
Applying a bandage	8	8			Included
Counseling on signs of a postinjection flare	8	7			Included
Counseling on signs of a septic joint	8	8			Included
Icing the joint	8	4	6	5	Included
Resting the joint for 2 days	8	3	6	4	Included
Avoid strenuous exercise for 3-5 days	8	3	6	4	Included
Advising to self-monitor for pain relief and duration of pain relief	8	6	6	5	Included
Avoid submerging the joint for 48 hours	8	3	6	2	Excluded
Appropriate handling and disposal of sharps	8	8			Included

[a]Simplified to “Trainee gathers all pertinent supplies” during the focus group.

Workshop Development and Implementation

We designed a flipped classroom workshop to teach the procedures of knee and shoulder arthrocentesis to IM and physical medicine and rehabilitation (PM&R) residents at WUSM. IM residents participated in the workshop during their training program’s outpatient musculoskeletal didactic series near the end of their first postgraduate year in April and May 2020 and in April and May 2021, while workshops for PM&R residents occurred in September and October 2021 at the beginning of their second postgraduate year. Trainees from both specialties were believed to have commensurate arthrocentesis skills based on the timing of the workshop with their level of training.

Copyright © 2023 Murray et al. This is an open-access publication distributed under the terms of the Creative Commons Attribution license.

Table 2. Modified Delphi Results for Knee Arthrocentesis Procedural Checklist Items

Item	Round 1		Round 2		Included or Excluded
	Total Votes	No. of Votes to Include Item	Total Votes	No. of Votes to Include Item	
Trainee appropriately advises positioning the patient for knee injection:					
By having them seated, with their knee at 90 degrees of flexion	6	5			Excluded[a]
By having them supine, with roughly 20 degrees of knee flexion	6	6			Included
Trainee appropriately advises positioning the patient for a supine medial/lateral knee injection:[a]					
Patellar margins	6	6			Included
Patellar tendon	6	1			Excluded
Tibial condyles	6	2			Excluded
Femoral condyles	6	2			Excluded
To maintain sterility, trainee uses:					
Iodine or chlorhexidine	6	6			Included
Use of nonsterile gloves	6	2			Excluded
No-touch technique	6	5			Included
Regarding procedural technique, trainee:					
Initiates a timeout	6	6			Included
Uses lidocaine along the trajectory of their approach	6	5			Included
Approaches roughly 1 cm superolateral from corner of patella, directed towards midpoint	6	5			Excluded
Inserts the needle medial or lateral to patellar tendon while the patient is sitting	5	5			Included
Inserts the needle below the patellar margin, roughly one-third from the superior patellar edge	6	6			Excluded

[a]The focus group decided to focus on the supine medial/lateral approach.

Table 3. Modified Delphi Results for Shoulder Arthrocentesis Procedural Checklist Items

Item	Round 1		Round 2		Included or Excluded
	Total Votes	No. of Votes to Include Item	Total Votes	No. of Votes to Include Item	
Trainee appropriately advises positioning the patient for posterior glenohumeral injection:					
By having them adduct the arm to their side	6	3	5	4	Included
By having them hug a pillow to their chest	6	3	5	1	Excluded
Trainee appropriately advises positioning the patient for subacromial bursal injection:					
By having them hang their arm by their side	6	3	5	5	Included
By having them with their arm supported by their side	6	3	5	0	Excluded
Trainee appropriately identifies landmarks for a subacromial bursal injection:					
Posterior scapular spine	6	2			Excluded
Humeral head	6	2			Excluded
Acromion	6	5			Included
Trainee appropriately identifies landmarks for a glenohumeral joint injection:					
Posterior scapular spine	6	1			Excluded
Humeral head	6	6			Included
Acromion	6	6			Included
To maintain sterility, trainee uses:					
Iodine or chlorhexidine	6	6			Included
Use of sterile gloves	6	2			Excluded
No-touch technique	6	5			Included
Regarding procedural technique, trainee:					
Initiates a timeout	6	6			Included
Uses lidocaine along projected trajectory	6	2			Excluded
Posterior glenohumeral approach—1-2 cm below the posterior acromion and into the glenohumeral groove, towards the acromion process	6	3	5	3	Included
Posterior glenohumeral approach—direct needle into glenohumeral groove and into the glenohumeral joint	6	3	5	2	Excluded
Subacromial bursa injection—direct the needle below the acromion and above the humeral head	6	5			Included
Subacromial bursa injection—posterolateral approach 2-3 cm inferior to the acromion, with a slight angle upwards	6	1			Excluded

Copyright © 2023 Murray et al. This is an open-access publication distributed under the terms of the Creative Commons Attribution license.

The protocols for this study were reviewed and approved by the WUSM Institutional Review Board (reference number 202004135, May 14, 2020) and classified as exempt.

We created three instructional videos on preprocedural, procedural, and postprocedural arthrocentesis care (Appendices E-G), which trainees watched before the workshop. The first video reviewed the indications, contraindications, risks, alternatives, equipment, and postprocedural guidance of knee and shoulder arthrocentesis. The other two videos demonstrated the steps of performing knee and shoulder arthrocentesis, focusing on the anterior, medial, and lateral approaches of knee arthrocentesis and the posterior glenohumeral, lateral subacromial, and posterolateral subacromial shoulder arthrocentesis.

The in-person workshop lasted 75 minutes and included three stations (Appendix H). Trainees practiced performing arthrocentesis in small groups with no greater than a 2:1 trainee-to-facilitator ratio. We created visual aids (Appendices I-L), provided anatomic models, and used knee and shoulder arthrocentesis simulation models (Sawbones) during the workshops.

After residents had practiced the arthrocentesis techniques, we assessed their knee and shoulder arthrocentesis skills in two SASs. During the SASs, facilitators conveyed instructions to the trainees and assumed the identities of the simulated patients. Trainees interacted with the facilitator as they would a standardized patient for any verbal components of the SASs and demonstrated procedural steps on the simulation model.

Collection of Validity Evidence for Assessment Tools

Cook and Hatala's description of Messick's validity framework structured our approach to gathering validity evidence for our assessment tools.[17] First, we created four videos (Appendices M-P) that represented staged knee and shoulder arthrocentesis performances, including common errors and successes observed during a workshop pilot in 2020. Our multidisciplinary team implemented frame-of-reference training, using the standard for minimal proficiency described below (in the Results section entitled Defining Proficiency) and practicing scoring SASs depicted in the videos.[18]

Second, facilitators scored participants' SAS performances using the developed assessment tools. Sometimes, two facilitators graded one trainee's performance simultaneously without referencing each other's scores. Additionally, facilitators scored the residents' performance of knee and shoulder arthrocentesis against a modified version of the Global Rating Index for Technical Skills (GRITS), a global rating scale measuring proficiency in procedural skills in the surgical setting.[19] We omitted components specific to surgery, a process used previously for collecting validity evidence for procedural assessment tools.[20] We used these data to analyze interrater reliability and relational validity.

Workshop Evaluation

To evaluate the outcomes of our workshop, we first determined how many participants met the minimum threshold for competence. Second, we measured the change in residents' self-perceived ability to perform knee and shoulder arthrocentesis through retrospective pre-post Likert scales (Appendix Q).[19,20] Likert-type questions probed for the self-perceived ability to list the indications, contraindications, risks, and benefits of knee and shoulder arthrocentesis; prepare to perform knee and shoulder arthrocentesis; advance the arthrocentesis needle; and provide postprocedural care instructions. We presented learners with QR codes for accessing evaluation surveys at the end of the workshop and provided time for survey completion to enhance the response rate. Lastly, to estimate the workshop's potential effect on clinical practice, we calculated the number of arthrocentesis procedures performed in the resident primary care clinic and how many different residents performed them before and after the workshop.

Statistical Methods

We calculated Krippendorff's alpha to measure the interrater reliability of the total scores on the knee and shoulder arthrocentesis assessment tools. We set the standard of Krippendorff's alpha greater than .70 a priori, in keeping with statistical standards.[21] Power calculations indicated that we needed at least 17 knee and 17 shoulder SASs scored by two facilitators to reach this standard.[22] All observations were used to compute the Pearson correlation between our novel assessment tools' total scores and the modified GRITS scores. When multiple facilitators assessed the same resident, the mean of the scores was used.

We used the Wilcoxon signed rank test to compare retrospective pre-post Likert-scale data obtained from the postworkshop evaluation survey. For measures explicitly related to knee arthrocentesis skills, participants were compared based on whether they had previously completed a knee arthrocentesis procedure. For measures explicitly related to shoulder arthrocentesis skills, participants were compared based on whether they had previously completed a shoulder arthrocentesis procedure. For more general measures applying to knee and shoulder arthrocentesis, participants were separated by whether

Copyright © 2023 Murray et al. This is an open-access publication distributed under the terms of the Creative Commons Attribution license.

they had previously completed either a knee or shoulder arthrocentesis procedure. We used chi-square analysis to compare the number of arthrocenteses completed in the IM residents' primary care clinic pre- and postworkshop. All analyses were conducted in R (version 4.1.0; R Core Team).

Results

Defining Proficiency

We defined proficiency in knee and shoulder arthrocentesis as the ability to perform major and minor checklist items without errors that would compromise the integrity of the procedure or increase the patient's risk of complications. Proficient performance did not require accessing the joint or bursal space because sometimes imaging guidance is needed during patient care to accomplish this task and because the decision to appropriately abort a procedure is a form of competence. Competent learners would demonstrate a general awareness of the procedure's steps. They could include a few noncritical mistakes or major mistakes that were corrected during the procedure, as well as mild-to-moderate procedural hesitancy. Proficient learners would maintain a reasonable line of communication with the patient throughout the knee or shoulder arthrocentesis. The median total assessment score (checklist total + global rating score) of borderline SAS performances set the cutoff score for proficiency as 21 of 39 points (range: 16-25 points) for knee arthrocentesis and 23 of 37 points (range: 19-26 points) for shoulder arthrocentesis.

Additional Validity Evidence

The Pearson's correlations between the modified GRITS and our novel assessment tools were $r(46) = .758$, $p < .001$, for knee and $r(63) = .771$, $p < .001$, for shoulder, demonstrating strong relational validity of our tools. Multiple facilitators assessed 26 (54%) knee SASs and 26 (40%) shoulder SASs, producing strong interrater reliability scores (knee Krippendorff's $\alpha = .82$, shoulder Krippendorff's $\alpha = .77$).

Workshop Evaluation

In total, 113 residents (52 IM residents in 2020, 53 IM residents in 2021, eight PM&R residents in 2021) participated in the workshop. We collected data from 48 knee and 65 shoulder SASs as validity evidence during the 2020 and 2021 workshops. The number of SASs used for statistical analysis represents the subset of workshop participants who were assessed by members of the research team. All documented knee arthrocentesis SAS performances met the minimum score for competence, achieving at least 21 points (median total score: 35 points, range: 26-39 points) on the knee arthrocentesis assessment tool. All but one resident met the minimum score for competence on the shoulder arthrocentesis SAS (median score: 31 points, range: 20-38 points), with one resident earning a total assessment score of 20 points. All others achieved greater than 23 points.

We invited 61 residents to complete the evaluation survey after workshops held in 2021; 52 (85%) participated, 46 from IM and six from PM&R. Analysis of survey responses showed that residents without prior arthrocentesis experience reported a significant increase in their ability to obtain informed consent, conduct ancillary steps in the procedure, and provide patients with instructions for postarthrocentesis care ($p < .001$ in all three categories; Table 4). The ancillary steps defined on the survey included listing the needed materials, performing a timeout, positioning the patient, sterilizing the site, and maintaining a no-touch technique. Regardless of prior arthrocentesis experience, all participants reported increased skill levels in identifying knee arthrocentesis anatomic landmarks (previous experience $p = .01$, no experience $p < .001$) and needle driving technique (prior experience $p = .01$, no experience $p < .001$). In contrast, only those without previous experience reported enhanced skill levels in identifying shoulder arthrocentesis anatomic landmarks and needle driving technique ($p < .001$ for both; Table 4). In addition, all participants indicated they would be more likely to offer knee and shoulder arthrocentesis to their patients after the workshop (prior experience $p = .002$, no experience $p < .001$).

The number of arthrocenteses performed in the IM residents' primary care clinic significantly increased from 15 during the 2019-2020 academic year to 36 during the 2021-2022 academic year ($\chi^2 = 8.647$, $p = .003$). The number of IM residents providing arthrocentesis during patient care increased from 10 (19%) during the 2019-2020 academic year to 16 (30%) during the 2020-2021 academic year, approaching statistical significance ($\chi^2 = 3.33$, $p = .07$).

Discussion

We present educational materials and assessment tools for teaching IM and PM&R residents the procedures of knee and shoulder arthrocentesis. Using a multidisciplinary approach, we defined the knowledge and skills needed to perform these procedures and incorporated them into hands-on workshops grounded in behavioral learning theory. These workshops enhanced residents' procedural skills and patient care practice regardless of their previous experience performing arthrocentesis. Furthermore, we collected strong evidence to support using our assessment tools when measuring knee and shoulder arthrocentesis proficiency during simulated scenarios.

Copyright © 2023 Murray et al. This is an open-access publication distributed under the terms of the Creative Commons Attribution license.

Table 4. Wilcoxon Signed Rank Test Comparing Self-Reported Skills Before and After the Workshop

Measure	Previous Experience With Knee or Shoulder Injections	N	Median[a] Preworkshop	Median[a] Postworkshop	p	Standard Normal Distribution (z)	Effect Size (r)
Overall injection skills							
List risks, benefits, indications, and contraindications during informed consent	Yes	17	3	4	.08	1.732	.42
	No	35	3	4	<.001	4.221	.71
Conduct ancillary procedures	Yes	17	3	4	.04	2.111	.51
	No	35	3	4	<.001	4.101	.69
Provide instructions for postprocedural care	Yes	17	3	4	.01	2.489	.60
	No	35	3	4	<.001	4.181	.71
Knee injection skills							
Identify knee injection landmarks	Yes	14	3	4	.01	2.530	.68
	No	38	2	4	<.001	4.300	.70
Knee injection needle insertion	Yes	14	3	4	.01	2.450	.65
	No	38	2	4	<.001	4.452	.72
Shoulder injection skills							
Identify shoulder injection landmarks	Yes	10	3	4	.07	1.841	.58
	No	42	2	4	<.001	4.788	.74
Shoulder injection needle insertion	Yes	10	3	3.5	.04	2.070	.65
	No	42	2.5	4	<.001	4.757	.73
Likelihood of offering knee and shoulder injections to patients	Yes	17	2	4	.002	3.025	.73
	No	35	1	3	<.001	5.023	.85

[a]Represents the median Likert-scale response (1 = *very poor,* 2 = *poor,* 3 = *good,* 4 = *very good*).

The design of our flipped classroom workshop provides flexibility for learning and efficiency for teaching. We created three videos for residents to review before a 75-minute hands-on session. The use of prework increased learner independence, as residents could watch the videos at times, places, and speeds most convenient for them. This approach also maximized the use of limited teaching resources, namely, the time experienced clinicians volunteered to supervise residents' hands-on practice. Other published curricula require from 90 minutes to several days of dedicated in-class instruction.[4,8,12,13,23,24] For example, Bretagne and colleagues used simulation models, cadavers, and 3 half-days of in-person instruction to improve learners' confidence and theoretical knowledge of arthrocentesis.[13] In comparison, our flipped classroom workshop provided learners with an effective educational experience in 75 minutes. Additionally, the materials can be presented en bloc—first showing the videos to a group of learners before leading them in the guided practice of knee and shoulder arthrocentesis—by educators who prefer a different workshop structure.

The content of our materials is generalizable to learners across specialties. We formed a multidisciplinary group of educators from IM, physiatry, and rheumatology and completed a modified Delphi to capture the group's viewpoints in a standardized way. Furthermore, we studied our materials within two residency programs, IM and PM&R. These methods enhanced the generalizability, reliability, and validity of the materials we created.

Our workshop uniquely incorporates assessment strategies and tools in its materials, whereas previous publications provide either instructional materials or assessment tools.[4,5,7-9,11-13,23,24] Our strategy reinforces the methods of curricular design for medical education.[25] In addition, our assessment tools generated strong evidence for reliability and validity, whereas previously published arthrocentesis assessment tools have considered only content validity.[4,9,11-13,23] Herein, we provide educators with materials to train their team of assessors to achieve similarly valid and reliable results.[13]

Our methods limit the application of our curricular and assessment materials. First, the modified Delphi method remains subject to error. The results may have changed if other experts had participated and submitted different skills, such as learning how to exchange syringes during the procedure. Furthermore, we selected an agreement threshold of four of the six participants (67%) because it was similar to the standard of 70%. The typical standard of 70% would necessitate consensus among seven of the eight (88%) participants, a level of agreement higher than is commonly utilized in Delphi methodology.[15] Second, to define proficiency in knee and shoulder arthrocentesis, we used the modified borderline-group method, which has been shown to be less precise than the borderline regression method.[26] Reduced precision may be evident in the different cutoff scores for knee and shoulder arthrocentesis. Third, the simulated environment limits learners' exposure to anatomic variations and interferes with applying our assessment tools to workplace-

Copyright © 2023 Murray et al. This is an open-access publication distributed under the terms of the Creative Commons Attribution license.

based observations. Fourth, IM and PM&R residents from a single institution participated in the workshop, restricting generalizability to other disciplines and locations.

Future initiatives should assess the performance of the arthrocentesis assessment tools in the patient care environment. This could provide validity evidence for their use in measuring procedural proficiency during workplace-based assessment and create opportunities to correlate SAS performance with procedural skills in patient care.

In summary, we offer educational materials and assessment tools for teaching IM and PM&R residents how to perform knee and shoulder arthrocentesis. Our work publishes the first assessment tools with strong validity evidence for these procedures. In addition, it demonstrates how these educational initiatives rejuvenate the provision of arthrocentesis in the clinical environment, enhancing the care of patients with musculoskeletal conditions.

Appendices

A. Shoulder Checklist and GRS.docx
B. Knee Checklist and GRS.docx
C. Sim Case 1 - Knee.docx
D. Sim Case 2 - Shoulder.docx
E. Training 1 - Intro.mp4
F. Training 2 - Knee.mp4
G. Training 3 - Shoulder.mp4
H. Workshop Flow.docx
I. Visual Aid - Knee 1.pdf
J. Visual Aid - Knee 2.pdf
K. Visual Aid - Shoulder.pdf
L. Injection Workflow Visual.pdf
M. Assessor Training - Knee 1.mp4
N. Assessor Training - Knee 2.mp4
O. Assessor Training - Shoulder 1.mp4
P. Assessor Training - Shoulder 2.mp4
Q. Postworkshop Survey.docx

All appendices are peer reviewed as integral parts of the Original Publication.

Jacob Murray, DO: Fellow, Division of Rheumatology, Washington University School of Medicine in St. Louis; ORCID: https://orcid.org/0000-0002-6717-9899

Andrea Ramirez-Gomez, MD: Clinical Associate of Medicine, Section of Rheumatology, University of Chicago Pritzker School of Medicine

Mike Cahill, PhD: Research Scientist and Data Analyst, Center for Integrative Research on Cognition, Learning and Education, Washington University in St. Louis

Amber Deptola, MD: Attending Physician, Internal Medicine, Norton Medical Group; ORCID: https://orcid.org/0000-0002-3808-2780

Colin Diffie, MD: Assistant Professor of Medicine, Division of Rheumatology, Washington University School of Medicine in St. Louis

Peter McDonnell, MD: Instructor in Medicine, Division of General Medicine, Washington University School of Medicine in St. Louis

John P. Metzler, MD: Associate Professor of Orthopedic Surgery, Division of Physical Medicine and Rehabilitation, Washington University School of Medicine in St. Louis; ORCID: https://orcid.org/0000-0003-0248-3758

Nathan P. Olafsen, MD: Assistant Professor of Orthopedic Surgery, Division of Physical Medicine and Rehabilitation, Washington University School of Medicine in St. Louis; ORCID: https://orcid.org/0000-0003-1485-232X

Lisa Zickuhr, MD, MHPE: Assistant Professor of Medicine, Division of Rheumatology, Washington University School of Medicine in St. Louis; ORCID: https://orcid.org/0000-0002-8260-9789

Acknowledgments
We thank Dominique Cosco, MD, and Maria Gonzalez-Mayda, MD, for participating in the modified Delphi.

Disclosures
None to report.

Funding/Support
This work was funded by the Small Grants Program of the Medical Education Research Unit and the Academy of Educators, Washington University School of Medicine in St. Louis.

Ethical Approval
The Washington University School of Medicine in St. Louis Institutional Review Board deemed further review of this project not necessary.

References

1. The burden of musculoskeletal diseases in the United States: prevalence, societal and economic cost. United States Bone and Joint Initiative. Accessed January 1, 2020. https://www.boneandjointburden.org/
2. Wittich CM, Ficalora RD, Mason TG, Beckman TJ. Musculoskeletal injection. *Mayo Clin Proc*. 2009;84(9):831-837. https://doi.org/10.4065/84.9.831
3. About medical procedures required during residency training. American College of Physicians. Accessed October 20, 2022. https://www.acponline.org/about-acp/about-internal-

Copyright © 2023 Murray et al. This is an open-access publication distributed under the terms of the Creative Commons Attribution license.

medicine/career-paths/residency-career-counseling/medical-board-exam-how-to-prepare-for-im-board-certification/the-board-certification-process/about-medical-procedures-required-during-residency-training

4. Vogelgesang SA, Karplus TM, Kreiter CD. An instructional program to facilitate teaching joint/soft-tissue injection and aspiration. *J Gen Intern Med*. 2002;17(6):441-445. https://doi.org/10.1046/j.1525-1497.2002.10310.x
5. Berman JR, Ben-Artzi A, Fisher MC, Bass AR, Pillinger MH. A comparison of arthrocentesis teaching tools: cadavers, synthetic joint models, and the relative utility of different educational modalities in improving trainees' comfort with procedures. *J Clin Rheumatol*. 2012;18(4):175-179. https://doi.org/10.1097/RHU.0b013e318258259e
6. Houston TK, Connors RL, Cutler N, Nidiry MA. A primary care musculoskeletal clinic for residents: success and sustainability. *J Gen Intern Med*. 2004;19(5):524-529. https://doi.org/10.1111/j.1525-1497.2004.30173.x
7. Barilla-LaBarca ML, Tsang JC, Goldsmith M, Furie R. Design, implementation, and outcome of a hands-on arthrocentesis workshop. *J Clin Rheumatol*. 2009;15(6):275-279. https://doi.org/10.1097/RHU.0b013e3181b68a62
8. Lenhard A, Moallem M, Marrie RA, Becker J, Garland A. An intervention to improve procedure education for internal medicine residents. *J Gen Intern Med*. 2008;23(3):288-293. https://doi.org/10.1007/s11606-008-0513-4
9. Cuccurullo S, Brown D, Petagna AM, Platt H, Strax TE. Musculoskeletal injection skills competency in physical medicine and rehabilitation residents: a method for development and assessment. *Am J Phys Med Rehabil*. 2004;83(6):479-485. https://doi.org/10.1097/00002060-200406000-00013
10. Jaffe E, Barker A, Battistone MJ. Measuring cognitive load during arthrocentesis training: our initial experience. *Arthritis Rheumatol*. 2018;70(suppl 9):abstract 858. https://acrabstracts.org/abstract/measuring-cognitive-load-during-arthrocentesis-training-our-initial-experience/
11. Preisner R, Jasti H, Elnicki M, Jeong K. Impact of web-based review on long-term retention of simulation-acquired knee and shoulder aspiration and injection skills. *J Grad Med Educ*. 2012;4(4):460-466. https://doi.org/10.4300/JGME-D-11-00239.1
12. Seifert MK, Holt CT, Haskins A, Dexter W. Improving internal medicine resident comfort with shoulder and knee joint injections using an injection workshop. *MedEdPORTAL*. 2020;16:10979. https://doi.org/10.15766/mep_2374-8265.10979
13. Bretagne V, Delapierre A, Cerasuolo D, Bellot A, Marcelli C, Guillois B. Randomized controlled study of a training program for knee and shoulder arthrocentesis on procedural simulators with assessment on cadavers. *ACR Open Rheumatol*. 2022;4(4): 312-321. https://doi.org/10.1002/acr2.11400
14. Skinner BF. *About Behaviourism*. Jonathan Cape; 1974.
15. Humphrey-Murto S, Varpio L, Gonsalves C, Wood TJ. Using consensus group methods such as Delphi and nominal group in medical education research. *Med Teach*. 2017;39(1):14-19. https://doi.org/10.1080/0142159X.2017.1245856
16. De Champlain AF. Ensuring that the competent are truly competent: an overview of common methods and procedures used to set standards on high-stakes examinations. *J Vet Med Educ*. 2004;31(1):62-66. https://doi.org/10.3138/jvme.31.1.62
17. Cook DA, Hatala R. Validation of educational assessments: a primer for simulation and beyond. *Adv Simul (Lond)*. 2016;1:31. https://doi.org/10.1186/s41077-016-0033-y
18. Kim S, Copeland L, Cohen E, Galt J, Terregino CA, Pradhan A. Frame-of-reference training for students: promoting a shared mental model for clerkship performance with an online, interactive training module. *J Gen Intern Med*. 2022;37(6): 1575-1577. https://doi.org/10.1007/s11606-021-06827-0
19. Doyle JD, Webber EM, Sidhu RS. A universal global rating scale for the evaluation of technical skills in the operating room. *Am J Surg*. 2007;193(5):551-555. https://doi.org/10.1016/j.amjsurg.2007.02.003
20. Hartman N, Wittler M, Askew K, Hiestand B, Manthey D. Validation of a performance checklist for ultrasound-guided internal jugular central lines for use in procedural instruction and assessment. *Postgrad Med J*. 2017;93(1096):67-70. https://doi.org/10.1136/postgradmedj-2015-133632
21. Krippendorff K. Reliability in content analysis: some common misconceptions and recommendations. *Hum Commun Res*. 2004;30(3):411-433. https://doi.org/10.1111/j.1468-2958.2004.tb00738.x
22. Bujang MA, Baharum N. A simplified guide to determination of sample size requirements for estimating the value of intraclass correlation coefficient: a review. *Arch Orofac Sci*. 2017;12(1): 1-11.
23. Wilcox T, Oyler J, Harada C, Utset T. Musculoskeletal exam and joint injection training for internal medicine residents. *J Gen Intern Med*. 2006;21(5):521-523. https://doi.org/10.1111/j.1525-1497.2006.00442.x
24. Jolly M, Hill A, Mataria M, Agarwal S. Influence of an interactive joint model injection workshop on physicians' musculoskeletal procedural skills. *J Rheumatol*. 2007;34(7):1576-1579.
25. Thomas PA, Kern DE, Hughes MT, Chen BY, eds. *Curriculum Development for Medical Education: A Six-Step Approach.* 3rd ed. Johns Hopkins University Press; 2016.
26. Wood TJ, Humphrey-Murto SM, Norman GR. Standard setting in a small scale OSCE: a comparison of the modified borderline-group method and the borderline regression method. *Adv Health Sci Educ Theory Pract*. 2006;11(2):115-122. https://doi.org/10.1007/s10459-005-7853-1

Received: July 26, 2022
Accepted: January 2, 2023
Published: April 13, 2023

Copyright © 2023 Murray et al. This is an open-access publication distributed under the terms of the Creative Commons Attribution license.
