# Supplementary material for: Workshop, Assessment, and Validity Evidence for Tools Measuring Performance of Knee and Shoulder Arthrocentesis
Source: MedEdPORTAL. 2023 Apr 13;19:11309. doi: 10.15766/mep_2374-8265.11309 (PMC10101652; doi:10.15766/mep_2374-8265.11309)
Supplement: Supplementary file 1 — Shoulder Checklist and GRS.docxKnee Checklist and GRS.docxSim Case 1 - Knee.docxSim Case 2 - Shoulder.docxTraining 1 - Intro.mp4Training 2 - Knee.mp4Training 3 - Shoulder.mp4Workshop Flow.docxVisual Aid - Knee 1.pdfVisual Aid - Knee 2.pdfVisual Aid - Shoulder.pdfInjection Workflow Visual.pdfAssessor Training - Knee 1.mp4Assessor Training - Knee 2.mp4Assessor Training - Shoulder 1.mp4Assessor Training - Shoulder 2.mp4Postworkshop Survey.docx [file mep_2374-8265.11309-s001.zip › L. Injection Workflow Visual.pdf]

12 Steps for Knee and Shoulder Injections

1. Consent the patient

Explain the procedure / Indications and benefits

Inflammatory arthritis:
RA, spondyloarthropathies, crystalline arthritis
Non inflammatory arthritis:
Osteoarthritis
Periarticular/soft tissue:
Bursitis, epicondylitis, tenosynovitis, carpal tunnel

1. Consent the patient

Contraindications:

- Broken skin at the injection site
- Overlying skin infection
- Intra articular fracture
- Known hypersensitivity to intra-articular agent
- Prosthetic joint (these should be done by Ortho)
- Anticoagulation is NOT a contraindication

1. Consent the patient

Risks

Joint
Post injection flare (5%), joint infection (1/3000), osteonecrosis (0.1-3%), cartilage damage
Surrounding tissue
Tendon rupture, neurovascular damage, skin atrophy/ depigmentation
Systemic
Facial flushing, vasovagal reaction, hypersensitivity reaction, transient hyperglycemia, bleeding

1. Consent the patient

Alternatives

Inflammatory arthritis:
Oral or intramuscular steroids
Non inflammatory arthritis:
PT, oral or topical NSAIDs, weight loss, joint replacement
Periarticular/soft tissue:
PT, splinting, rest

2.Pick your approach

- **Knee**: Medial, lateral, anterior, superior, suprapatellar
- **Subacromial bursa**: lateral, posterior
- **Glenohumeral**: posterior

3.Position the patient

- Knee supine: knee at 20°
- Knee sitting: knee dangling
- SAC bursa: arm dangling
- GH: abducted, supported

4.Identify your landmarks

5.Mark injection site

6.Gather supplies

7.Sterilize site

Chlorhexidine (back and forth technique, NOT circular)
No-touch technique (sterile gloves are not needed)

8.Perform time out

9.Access the joint

Indication: Arthrocentesis (fluid removal):
Topical anesthesia followed by injectable anesthesia with 1-2% lidocaine without epinephrine

9.Access the joint

Indication: Steroid injection:

- Topical anesthesia alone can be used
- Steroid options: Methylprednisolone acetate (MP)
- Triamcinolone acetonide (TM)

9. Access the joint

Steroid dosing varies with the structure injected.

Small (MCP, PIP, tendon sheath): MP 10-20 mg, TM 8-10 mg
Medium (wrist, ankle, elbow): MP 40-60mg, TM 20-30 mg
Large (knee, shoulder, hip): MP 40-80mg, TM 20-40mg

10.Manage sharps

11.Care for injection site

Apply pressure
Apply bandage

12.Provide anticipatory guidance

- Rest/Ice the joint
- Warning signs and instructions for septic joint
- Post-injection flare
- Possible systemic side effects: Diabetes management, drug-drug interactions
