# Supplementary material for: Workshop, Assessment, and Validity Evidence for Tools Measuring Performance of Knee and Shoulder Arthrocentesis
Source: MedEdPORTAL. 2023 Apr 13;19:11309. doi: 10.15766/mep_2374-8265.11309 (PMC10101652; doi:10.15766/mep_2374-8265.11309)
Supplement: Supplementary file 1 — Shoulder Checklist and GRS.docxKnee Checklist and GRS.docxSim Case 1 - Knee.docxSim Case 2 - Shoulder.docxTraining 1 - Intro.mp4Training 2 - Knee.mp4Training 3 - Shoulder.mp4Workshop Flow.docxVisual Aid - Knee 1.pdfVisual Aid - Knee 2.pdfVisual Aid - Shoulder.pdfInjection Workflow Visual.pdfAssessor Training - Knee 1.mp4Assessor Training - Knee 2.mp4Assessor Training - Shoulder 1.mp4Assessor Training - Shoulder 2.mp4Postworkshop Survey.docx [file mep_2374-8265.11309-s001.zip › Q. Postworkshop Survey.docx]

Post-Workshop Feedback Survey

Dear Resident,
  Congratulations on completing the instructional videos and workshop on knee and shoulder injections!  Please complete the following survey to give feedback on your experience.  Your responses are anonymous and voluntary.  They will be used to evaluate the utility of these modules and guide future improvements.  The survey should take you 2-5 minutes to complete. For questions, please contact [lead facilitator] at [lead facilitator email address].

Thank you,

[authors]

Question: Did you watch the injection videos?

- Yes (1)
- No (2)

Skip next question if you did not watch the videos.

Question: Use the scale to rank...

|  | Very Poor (1) | Poor (2) | Good (3) | Very Good (4) |
| --- | --- | --- | --- | --- |
| The **usefulness** of the knee and shoulder injection **videos** (1) |  |  |  |  |
| The **quality** of the knee and shoulder injection **videos** (2) |  |  |  |  |

Question: Use the scale to rank...

|  | Very Poor (1) | Poor (2) | Good (3) | Very Good (4) |
| --- | --- | --- | --- | --- |
| The **usefulness** of the knee and shoulder injection **workshop** (1) |  |  |  |  |
| The **quality** of the knee and shoulder injection **workshop** (2) |  |  |  |  |

Question: Please indicate your ability to perform the following tasks **before** completing the joint injection videos and workshop:

|  | Very Poor (1) | Poor (2) | Good (3) | Very Good (4) |
| --- | --- | --- | --- | --- |
| Include the indications, contraindications, risks, benefits, and alternatives for knee and shoulder injections in informed consent (1) |  |  |  |  |
| Conduct ancillary procedures involved in knee and shoulder injections, such as listing needed materials, performing a time-out, positioning the patient, sterilizing the site and maintaining no-touch technique (2) |  |  |  |  |
| Use anatomic landmarks to identify sites for **knee** injections (3) |  |  |  |  |
| Accurately insert and advance the needle during **knee** injections (4) |  |  |  |  |
| Use anatomic landmarks to identify sites for **shoulder** injections (5) |  |  |  |  |
| Accurately insert and advance the needle during **shoulder** injections (6) |  |  |  |  |
| Provide instructions for post-procedural care following joint injections (7) |  |  |  |  |

Question: Please indicate your ability to perform the following tasks **after** completing the joint injection videos and workshop:

|  | Very Poor (1) | Poor (2) | Good (3) | Very Good (4) |
| --- | --- | --- | --- | --- |
| Obtain informed consent that includes the indications, contraindications, risks, benefits, and alternatives for knee and shoulder injections (1) |  |  |  |  |
| Conduct ancillary procedures involved in knee and shoulder injections, such as listing needed materials, performing a time-out, positioning the patient, sterilizing the site and maintaining no-touch technique (2) |  |  |  |  |
| Provide instructions for post-procedural care following joint injections (3) |  |  |  |  |
| Use anatomic landmarks to identify sites for **knee** injections (4) |  |  |  |  |
| Accurately insert and advance the needle during **knee** injections (5) |  |  |  |  |
| Use anatomic landmarks to identify sites for **shoulder** injections (6) |  |  |  |  |
| Accurately insert and advance the needle during **shoulder** injections (7) |  |  |  |  |

Question: Did you have experience performing **knee** injections on patients before the workshop?

- Yes (1)
- No (2)

Question: Did you have experience performing **shoulder** injections on patients before the workshop?

- Yes (1)
- No (2)

Question: How likely were/are you to offer knee and shoulder injections to your patients...

|  | Unlikely (1) | Somewhat likely (2) | Likely (3) | Very Likely (4) |
| --- | --- | --- | --- | --- |
| **Before** the workshop (1) |  |  |  |  |
| **After** the workshop (2) |  |  |  |  |

Question: Please provide additional comments in the box below.

________________________________________________________________
