# Supplementary figures and images for: Workshop, Assessment, and Validity Evidence for Tools Measuring Performance of Knee and Shoulder Arthrocentesis
Source: MedEdPORTAL. 2023 Apr 13;19:11309. doi: 10.15766/mep_2374-8265.11309 (PMC10101652; doi:10.15766/mep_2374-8265.11309)

# Approaches to glenohumeral and subacromial injections

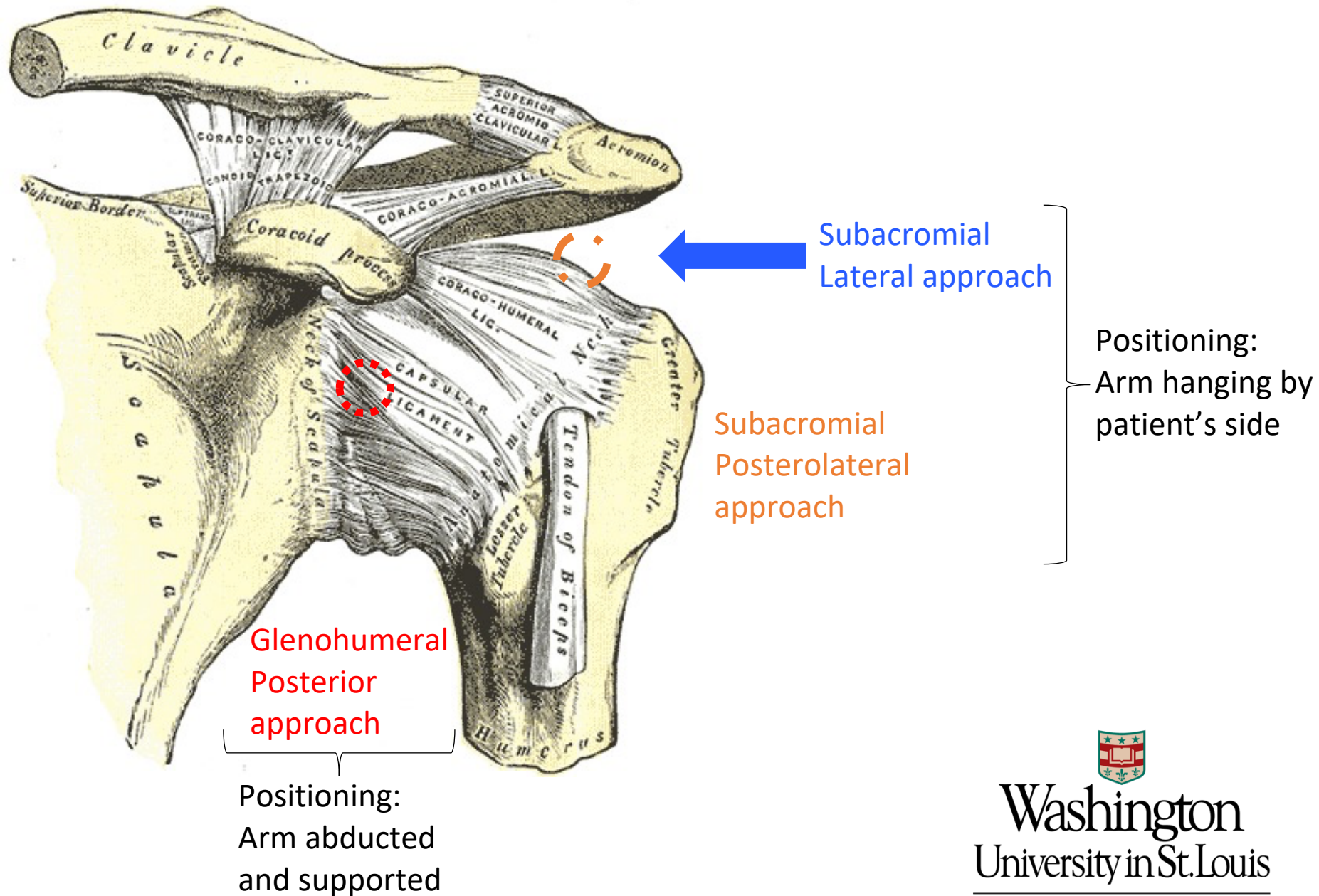

Supplement: Supplementary file 1 — Shoulder Checklist and GRS.docxKnee Checklist and GRS.docxSim Case 1 - Knee.docxSim Case 2 - Shoulder.docxTraining 1 - Intro.mp4Training 2 - Knee.mp4Training 3 - Shoulder.mp4Workshop Flow.docxVisual Aid - Knee 1.pdfVisual Aid - Knee 2.pdfVisual Aid - Shoulder.pdfInjection Workflow Visual.pdfAssessor Training - Knee 1.mp4Assessor Training - Knee 2.mp4Assessor Training - Shoulder 1.mp4Assessor Training - Shoulder 2.mp4Postworkshop Survey.docx [file mep_2374-8265.11309-s001.zip › K. Visual Aid - Shoulder.pdf]
